# Impact of Storage Conditions on Equine Fecal Inoculum for Estimating In Vitro Digestibility

**DOI:** 10.3390/ani11113195

**Published:** 2021-11-09

**Authors:** Delaney O’Donnell, Lacy Sukovaty, Gary Webb

**Affiliations:** Department of Animal Science, William H Darr College of Agriculture, Missouri State University, Springfield, MO 65897, USA; delaney.odonnell96@gmail.com (D.O.); GaryWebb@missouristate.edu (G.W.)

**Keywords:** equine, digestibility, microbial inoculum, in vitro, inoculum storage

## Abstract

**Simple Summary:**

Sample handling and storage may affect the fermentative capacity of fecal inoculum. The need to collect rectal grabs from individuals can be a limiting factor in utilizing fecal inoculum from very young or feral animals. This study evaluated the effect of storage conditions of equine feces on the viability of microbial inoculum used for in vitro equine digestibility trials. Pooled fecal material was stored anaerobically at 39 °C for 15 min (control), while aerobic samples were stored at 22 °C for 6 h (SC1), 3 °C for 6 h (SC2), and −18 °C for 24 h (SC3). Results supported that fecal material stored aerobically for six hours at 22 °C provided similar digestibility estimates compared to the control, while dry matter digestibility decreased by 3.86% at SC2 and by 4.08% at SC3.

**Abstract:**

This study evaluated the effect of storage conditions of equine fecal material on the viability of microbial inoculum used for in vitro equine digestibility trials. Pooled fecal material from three mature Quarter Horse geldings was stored at 39 °C anaerobically for 15 min (control), while aerobic samples were stored at 22 °C for 6 h (SC1), 3 °C for 6 h (SC2), and −18 °C for 24 h (SC3). Following storage, the feces were utilized to prepare microbial inoculum for the digestion of six different forages using the Daisy II Incubator. After incubation, DM, NDF, and ADF compositions were determined and used to calculate DMD, NDFD, and ADFD. Analysis using the OLS regression model for differences in DMD, NDFD, and ADFD across the storage conditions found significant interactions between the forage sample and the storage condition (*p* < 0.05). The results between the control and SC1, SC2, and SC3 were not different (*p* < 0.8). Fecal material stored aerobically for six hours at 22 °C provided similar digestibility estimates compared to the control, while DMD decreased by 3.86% in SC2 and by 4.08% in SC3.

## 1. Introduction

Previous studies have validated the use of equine feces as inoculum for in vitro equine digestibility studies using both gas production models and the ANKOM Daisy II Incubator [1,2,3]. Studies conducted by Lattimer et al. and Earing et al. both validated the use of microbial inoculum from equine feces for use in the batch culture Daisy II Incubator. These studies compared results from the Daisy II with in vivo digestibility trial results and found no significant differences between the two methods [2,3]. Earing et al. further validated the use of the Daisy II system with equine fecal inoculum, finding no significant differences between the Daisy II and in vivo results across four different diets. These studies in horses showed higher model fits of in vitro digestibility estimates to in vivo models from a 48 h incubation period than from 24 or 72 h [2,3]. A study by Tassone et al. on in vitro digestion in the Daisy II Incubator for donkeys noted that accuracy increased if incubation was extended to at least 60 h, whereas for horses, higher model fits are seen at 48 h of incubation [4]. However, both in vivo and in vitro results in that study ranked the experimental diets in the same order. These results highlight the importance of considering the differences in transit time and microbial populations between species of equine.

In vitro digestibility studies provide a faster, more accessible, and more cost-effective way of evaluating the digestibility of equine diets than traditional methods using in vivo techniques. Additionally, in vitro batch culture techniques allow for the simultaneous study of multiple feedstuffs with less labor than in vivo digestibility trials [5]. Previous studies have used fresh fecal material, collected via rectal palpation for microbial inoculum, that was transported to the laboratory in anaerobic conditions at 39 °C [1,2,3]. Modifying two-stage digestion methods from ruminant research for equine use would suggest a need to first predigest the feed to simulate pre-cecal digestion in the equine before using microbial inoculum to fermentatively digest the feed. However, research has shown that enzymatic predigestion of feed delayed the onset of fermentation and required longer periods of fermentation to fully digest the feed [6]. Enzymatic predigestion of feed for in vitro equine research is necessary to accurately estimate the digestibility of high-starch, low-fiber feeds, but not necessary for low-quality, high-fiber feeds such as hay which can instead be evaluated using a simpler one-step method using just microbial inoculum [6].

Microbial inoculum provides a major source of variation within in vitro systems, making the reduction in variability a key need for further use of these systems. With hindgut fermentation in equines, fecal microbial populations are likely to be more representative of the microbial population than those from ruminant species. Lowman et al. found that equine fecal material was suitable for use as microbial inoculum for in vitro gas production techniques, while Lattimer et al. and Earing et al. both validated the use of microbial inoculum from equine feces for use in the batch culture Daisy II Incubator [1,2,3]. Modern management practices of meal feeding result in relatively large amounts of feed moving through the digestive tract together, as opposed to the natural condition of a continuous flow of small amounts of feed. This change in transit pattern within the digestive tract effects the ability of researchers to use fecal material as inoculum for in vitro digestibility studies [7]. Microbial inoculum dilution and substrate hydration are other potential sources of variability, as substrate is hydrated when it reaches the cecum for fermentation. Franzan et al. found that inoculum dilution does not alter fermentation, and substrate hydration increases the fermentation process, increasing nutrient degradation [8]. With large variations in microbial communities attributed to diet, collection time, and variation between individuals, microbial inoculum and its handling provides variability between runs and experiments.

There is limited research on the effects of temperature and environment on the fermentative ability of the microbes present in equine feces used for in vitro systems [9]. Preserved inoculum sources would provide researchers with the ability to use inoculum from animals located further distances from their laboratory facilities and to transport inoculum in a variety of temperatures and environmental conditions, and it would aid in standardizing in vitro research procedures and results [9]. Knowledge of how storage conditions, either chilling or freezing, effect the viability of inoculum would provide the ability to use the same inoculum across multiple runs, reducing a source of variation.

The purpose of this study was to evaluate how exposure to different temperatures and environmental conditions effects the fermentative capacity of inoculum formed from equine feces through analysis of dry matter digestibility (DMD), neutral detergent fiber digestibility (NDFD), and acid detergent fiber digestibility (ADFD) between forages of differing chemical compositions.

## 2. Materials and Methods

### 2.1. Ethical Considerations

This study was performed in accordance with the guidelines of the Institutional Animal Care and Use Committee of Missouri State University IACUC (#19-021).

### 2.2. Fecal Samples

Fecal samples were collected from three mature Quarter Horse geldings, aged 7, 8, and 19 years, averaging 517 kg of body weight, from the Missouri State University herd. Two animals were housed in 3.6 × 7.3 m covered pens on limestone footing; one was housed in a 35 × 35 m covered arena on sand. The animals were fed twice a day at 0700 and 1700, remaining on their normal rations of 3.6 kg per day of a commercial grain mix (Easy Keeper Edge, MFA Inc., Columbia, MO, USA) and 7.3 kg per day of locally produced fescue grass hay with ad libitum access to water (Table 1).

Fecal samples were collected from the same three geldings on three separate days over a two-week period at 1100 h via rectal palpation. Immediately following collection, fecal samples were placed individually in sealed plastic bags with air expressed from the container, and stored at 39 °C in a pre-warmed cooler until they reached the laboratory for processing (5–10 min). In the laboratory, pooled fecal samples weighing 45 g were formed by weighing out 15 g of fecal material from each horse. Four storage conditions were evaluated. One pooled fecal sample was sealed anaerobically at 39 °C, processed quickly, and immediately used to form inoculum (control). The other three pooled samples were left in unsealed plastic bags providing an aerobic environment and stored at 22 °C for 6 h (SC1), 3 °C for 6 h (SC2), and −18 °C for 24 h (SC3) to simulate a variety of storage conditions. The 22 °C sample was stored on the counter in a climate-controlled laboratory; the 3 °C sample was placed in a standard household refrigerator in the laboratory; and the −18 °C sample was placed in a standard household freezer in the laboratory. This procedure was repeated for three replicates over a two-week period.

### 2.3. Forage Samples

Six forages representing a variety of chemical compositions were used (Table 2). Representative samples were taken from each of the five bales of hay and one bag of alfalfa cubes. Samples were ground to pass through a 1 mm screen using a Wiley mill. The dry matter of each forage was determined in the laboratory by drying the forage for 24 h in a 50 ± 2 °C oven. The dry matter was calculated as (dry sample weight (g)/starting sample weight (g)) × 100 = percentage of dry matter, using a Sartorius m-power scale +/− 0.0002 g.

F57 filter bags (ANKOM Technology) were used within the Daisy II Incubator for the determination of forage digestibility. Thirteen filter bags were immersed in acetone for five minutes and then air dried on a wire screen. The empty filter bags were labeled and weighed. Two filter bags were allocated to each of the six forage samples and filled with a weight of approximately 0.50 g of forage each. The final weights were then recorded before sealing the bags with a 120 V Impulse Heat Sealer (American International Electric, City of Industry, CA, USA). The digestion jar also contained one blank bag without any forage, which was weighed and sealed to act as a control for comparison to the varying levels of forage particle attachment in the other sample bags in the digestion jar.

### 2.4. In Vitro Digestibility

Procedures described by Lattimer et al. [2] were followed for the preparation of the microbial inoculum from feces. One pooled sample was processed immediately while the other three were stored as described in 2.2 Fecal Samples. The digestion jars with the SC1, SC2, and SC3 samples had delayed incubation compared to the control. Within the incubator, digestion jar addition and removal occurred with negligible temperature change within the incubator.

A buffer solution was made by combining 1333 mL of solution A (comprising KH_2_PO_4_ 10.0 g/L; MgSO_4_·7H_2_O 0.5 g/L; NaCl 0.5 g/L; CaCl_2_·2H_2_O 0.1 g/L and CH₄N₂O 0.5 g/L) with 267 mL of solution B (comprising Na_2_CO_3_ 15.0 g/L and Na_2_S·7H_2_O 1.0 g/L) at 39 °C and titrating to a pH of 6.8. After mixing, 1600 mL of buffer solution was added to each digestion jar and allowed to equilibrate at 39 °C in the Daisy II Incubator for at least 30 min while fecal inoculum was being collected and prepared. To form inoculum, each 45 g pooled fecal sample was individually placed into a blender containing 400 mL of distilled water, then purged with CO_2_ and blended for 15 s. Inoculum was strained through four layers of cheesecloth into a beaker to remove solid particles. Strained inoculum, measuring 400 mL, was poured into ANKOM digestion jars containing the forage-filled filter bags and 1600 mL of buffer solution for a total of 2000 mL of mixed inoculum and buffer. The digestion jars were purged with CO_2_ for 30 s before being closed and placed into the ANKOM Daisy II Incubator (ANKOM Technology, Macedon, NY, USA). The Daisy II Incubator contained four digestion jars prepared with fecal inoculum from each of the four storage conditions. Jars were incubated at 39.5 °C for 48 h. The digestion jars containing the SC1, SC2, and SC3 samples had delayed incubation start times compared to the control. Within the incubator, digestion jar addition and removal caused negligible temperature change. After 48 h, the filter bags were removed from the jar, rinsed in cool water, dried in a 50 °C oven for 24 h, and cooled to room temperature in an ANKOM MoistureStop Weigh Pouch (ANKOM X45, ANKOM Technology, Macedon, NY, USA), before being individually removed and reweighed to determine DMD. Further analysis was conducted using an ANKOM Fiber Analyzer (ANKOM2000, ANKOM Technology, Macedon, NY, USA) to sequentially determine residual NDF and ADF fractions within the digested forage samples. These residual fractions were then used to calculate NDFD and ADFD.

### 2.5. Statistical Analysis

The individual jars in the Daisy II Incubator all contained fecal inoculum from each of the four storage conditions along with duplicates of the six forage samples. Mean scores from these duplicates were calculated and used for analysis. The data from this 4 × 6 factorial design with three replicates conducted over a two-week period were analyzed by ordinary least squares (OLS) regression. The forages had different levels of neutral detergent fiber (NDF) and acid detergent fiber (ADF). We measured the levels of NDF and ADF to include as covariates in the analysis.

We constructed the following OLS regression model to evaluate the effects of storage condition and forage type on in vitro dry matter digestibility ($DMD_{i})$:

$$DMD_{i}=\alpha_{0}+\overset{j-1}{\underset{j=1}{\sum }}\beta_{j}T_{ji}+\overset{k-1}{\underset{k=1}{\sum }}\gamma_{k}F_{ki}+\beta \;NDF_{i}+\beta \;ADF_{i}+u_{i}$$

where the $\beta_{j}$ coefficients are the storage condition effects estimated as the differences between the control storage condition (39 °C) and the remaining experimental storage conditions based on the inclusion of the remaining $T_{j-1}\;$ storage condition dummy variables. There were $F_{k-1}\;$ dummy variables included in the model to account for the differences between the k different types of forage. The $\gamma_{k}$ coefficients represent the differences between each forage type included in the model and the baseline forage (alfalfa cubes), i.e., the forage fixed effects. The coefficients $NDF_{i}$ and $ADF_{i}$ are the marginal effects of NDF and ADF, respectively. The coefficients $\alpha_{o}$ and $\mu_{i}$ are the y-intercept of the regression and random error.

The results from the analysis are presented in Table 3. There are two models in Table 3; Model 1 does not include controls for NDFD and ADFD and Model 2 does. The coefficients for the each of the storage conditions and the forages represent the differences between control (39 °C) as the baseline storage condition and alfalfa cubes as the baseline forage.

We utilized the following ANOVA using PROC MIXED to evaluate differences in means of DMD, NDFD, and ADFD, with forage type and storage conditions as fixed effects and replicate as a random effect:

$$Y_{ijkl}=\mu +\propto_{i}+\beta_{j}+\delta_{k}+\epsilon_{ijkl}$$

where μ is the overall mean, $Y_{ijkl}$ is the measure of digestibility dependent on the fixed effects of *α_i_*, storage condition, *β_j_*, forage effect with *δ_k_*, replicate treated as random effect, and error term, $\epsilon_{ijkl}$. The significance level was set to *p* ≤ 0.05.

## 3. Results

**Figure 1:**
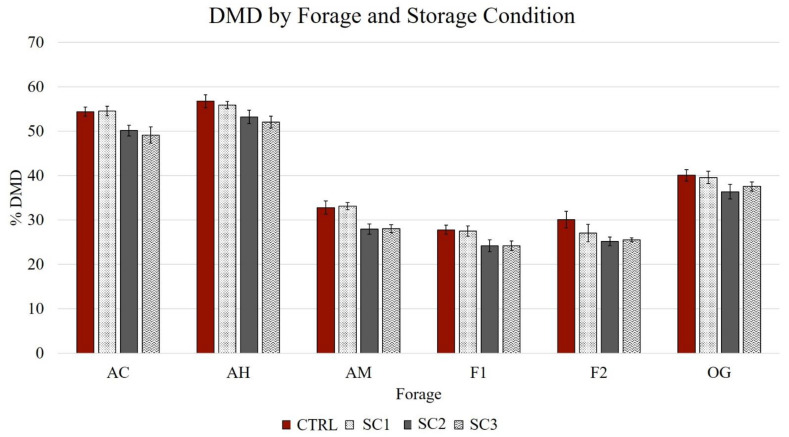
Equine dry matter digestibility of forage by microbial inoculum storage condition, grouped by forage type AC, alfalfa cubes; AH, alfalfa hay; AM, alfalfa mix; F1, fescue hay 1; F2, fescue hay 2; OG, orchardgrass hay.

In the OLS regression, differences were observed among the comparisons of the control to SC2 and the control to SC3; however, the result of the comparison between the control and SC1 was not significant (*p* < 0.05) (Table 3). The forage and replicate effects were significant. DMD, NDFD, and ADFD were negatively impacted when fecal inoculums were stored at SC2 or SC3. On average, DMD decreased by 3.86% ± 0.41 at SC2 and 4.08% ± 0.38 at SC3 (Figure 1).

## 4. Discussion

Analysis of DMD, NDFD, and ADFD showed that both the fresh fecal material held at 39 °C that was immediately used (control) and the fecal material held at 22 °C for 6 h in aerobic conditions (SC1) provided microbial inoculum that, when incubated with forage samples, yielded similar in vitro digestibility results.

Research on the preservation of equine fecal inoculum is limited, but it supports the conclusion from ruminant studies that the fermentative ability of preserved inoculum is substrate dependent. Freezing equine fecal material at −20 °C for 7 d reduced (*p* < 0.05) in vitro digestibility estimates for grass hay, but the same procedure did not affect estimates for alfalfa hay when using a gas production model. Further experiments showed that reducing the freezing time to 24, 48, or 72 h still significantly reduced digestibility estimates for grass hay but not for alfalfa hay [9]. Contrary to previous studies, the present study did not find that the reduction in in vitro digestibility estimates in SC2 and SC3 was substrate dependent.

In the current study, exposing fecal material to oxygen for up to 6 h did not significantly impact the fermentation capacity of the microbial population present in the fecal material, as no difference in in vitro digestibility was seen between the microbial inoculum formed from the control and that of SC1. Previous research has established that most bacterial species in the hindgut of the horse are anaerobic organisms that are oxygen-sensitive, and it would be expected that these bacteria would not remain viable when placed in an aerobic environment [10,11]. In one study, storage in anerobic conditions for 2 h was shown to significantly reduce cellulolytic bacteria populations in fecal material by over 90% when stored in 1 g samples [10]. In contrast, a recent publication by de Bustamante et al. found that aliquots of 2–3 g of feces stored in closed 118 mL containers for up to 6 h at room temperature prior to freezing did not affect the composition of the microbial population in equine feces, as compared to samples frozen immediately after collection. The same study also found that microbial populations were significantly changed by storage at ambient temperatures for periods longer than 6 h prior to freezing [12]. Samples from this de Bustamante et al. study were not used to form inoculum to digest forages.

In this study, freezing fecal material at −18 °C for 24 h (SC3) negatively impacted estimates of DMD, NDFD, and ADFD, suggesting that fecal material that has been frozen, either for storage in the laboratory or by natural weather conditions, is not a suitable replacement for fresh fecal material for the formation of microbial inoculum. Multiple studies from ruminant species have found that the use of frozen rumen fluid to form inoculum significantly reduces forage digestibility estimates [13,14,15,16]. Similar results have been reported by other authors, including Murray et al., who found the use of frozen equine feces to form inoculum significantly reduced the digestibility of all forage samples studied, with low-quality, high-ADF forages the most severely affected [9]. It is known that different functional groups of microorganisms living in the gastrointestinal tract have differing susceptibilities to changes from their normal environmental conditions. Amylases have been shown to be more resistant to freezing than either cellulases or xylanases [17]. Cellulolytic bacteria are primarily Gram-negative and are less resistant to freezing injury than Gram-positive bacteria. Cellulases are important for the digestion of low-quality forages, likely explaining the substrate-dependent effect of frozen inoculum on digestibility in earlier studies [9,18,19].

Unexpectedly, there was no difference observed between the two cold treatments of microbial inoculum. Refrigerator storage of fecal material at 3 °C for 6 h (SC2) negatively affected DMD, NDFD, and ADFD to the same degree as freezing. This contrasts with previous research that has found the short-term refrigeration of rumen fluid from both cattle and sheep for up to 6 h to be an acceptable storage technique [13,16,19]. To our knowledge, no other studies have evaluated the effect of short-term refrigeration storage on the fermentative capacity of microbial inoculum formed from equine feces. Microbial death is likely not the reason for the reduction in digestibility, as freezing at −18 °C should result in higher death rates than storage at 3 °C, and these higher death rates would result in a concurrent decrease in digestibility estimates [20]. It is possible that the reduction in DMD, NDFD, and ADFD that occurred between SC2 and SC3 was the result of microbial lag. Bacteria subjected to cold storage are known to have a lag time of several hours before they begin multiplying and producing enzymes at a normal rate [21].

The limitations of this study include that a wide range of temperatures were evaluated (39 °C, 22 °C, 3 °C, and −18 °C) in the storage conditions. Further research will be needed to determine more specific bounds for temperature and time limits for the storage of microbial inoculum between the tested storage temperatures of 22 °C and 3 °C. Inter-horse variation is a known source of inoculum variability; our study utilized a small, uniform equine population on a controlled diet. A wider range of NDF and ADF values of the forage substrates digested within the incubator may have revealed more differences between storage temperature and forage quality. Bacterial enumeration and post-fermentation pH measurements not utilized in this study would help in establishing the cause of the negative impacts on in vitro digestibility measures seen in the SC2 and SC3 samples.

As we refine our research techniques to involve less direct manipulation of research animals and many institutions encourage undergraduate research, validation of the use of stored fecal material for the formation of microbial inoculum for in vitro digestibility studies provides researchers with the ability to use inoculum from animals located at further distances from their laboratory facilities, to transport inoculum in a wider variety of temperatures and environmental conditions, and to explore the use of naturally deposited fecal material.

## 5. Conclusions

Our results indicate that short-term storage and transportation of equine feces for the formation of microbial inoculum for up to 6 h at 22 °C under aerobic conditions is possible when the use of fresh fecal material is not available. Both chilled (SC2) and frozen (SC3) fecal material provided digestibility estimates that were significantly lower than fresh inoculum, suggesting that cold-stored fecal material under aerobic conditions should not be used to form microbial inoculum for in vitro digestibility studies. Further studies will be needed to evaluate the effect of combinations of various temperatures, times, and humidity conditions on the survival of microbial populations and any influence of environmental contamination on naturally deposited feces for microbial inoculum.

## Figures and Tables

**Table 1:** Chemical composition (%) of concentrate (Easy Keeper Edge, MFA) and fescue hay.

Nutrient	DM	Ash	EE	ADF	NDF	NFC	CP
Concentrate	90.67	6.89	5.83	22.99	40.03	31.81	15.44
Fescue Hay	91.22	----	----	45.05	62.40	----	9.26

DM, dry matter; Ash, mineral matter; EE, —ether extract; ADF, acid detergent fiber; NDF, neutral detergent fiber; NFC, non-fibrous carbohydrates (= 100 − (CP + EE + NDF + Ash); CP, crude protein.

**Table 2:** Nutrient analysis of forages (% DM basis) with NDF and ADF determined by wet chemistry and CP determined by NIR spectrometry.

Forage	% DM	% NDF	% ADF	% CP
Alfalfa cubes	91.59	40.75	32.15	19.88
Alfalfa hay	92.01	45.71	31.93	19.60
Alfalfa-grass mix hay	91.53	61.27	42.71	11.57
Fescue hay 1	91.24	61.69	44.67	5.97
Fescue hay 2	91.22	62.40	45.05	9.26
Orchardgrass hay	91.59	53.85	40.29	13.76

DM, dry matter; NDF, neutral detergent fiber; ADF, acid detergent fiber; CP, crude protein.

**Table 3:** Storage conditions and dry matter digestibility.

	Model 1	Model 2
22 °C (SC1)	−0.50 (0.32)	−0.45 (0.33)
3 °C (SC2)	−4.05 *** (0.32)	−3.86 *** (0.41)
−18 °C (SC3)	−4.22 *** (0.31)	−4.08 *** (0.38)
Alfalfa Grass Mix	2.40 *** (0.38)	2.58 *** (0.46)
Alfalfa Hay	−21.59 *** (0.38)	−21.02 *** (1.06)
Fescue Hay 1	−26.16 *** (0.38)	−25.56 ***
Fescue Hay 2	−24.80 *** (0.39)	−24.24 *** (1.14)
Orchard Grass	−13.56 *** (0.39)	−13.29 *** (0.63)
NDFD		−0.07 (0.13)
ADFD		0.10 (0.14)
9/16 Replicate	−1.70 *** (0.27)	−1.67 *** (0.28)
9/23 Replicate	−3.88 *** (0.28)	−3.86 *** (0.29)
Constant	56.15 *** (0.37)	54.85 *** (2.57)
Adj. R^2^	0.99	0.99
Num. obs.	72	72

*** *p* < 0.01. OLS estimates with standard errors in parentheses. Baseline Storage condition is 39 °C (CTRL). Baseline forage is Alfalfa Cubes.

## Data Availability

The data presented in this study are available on request to the corresponding author.
